# Supplementary material for: Genetic Variation and Sensory Perception of a Pediatric Formulation of Ibuprofen: Can a Medicine Taste Too Good for Some?
Source: Int J Mol Sci. 2023 Aug 22;24(17):13050. doi: 10.3390/ijms241713050 (PMC10487938; doi:10.3390/ijms241713050)

SUPPLEMENTARY MATERIALS

Supplementary Figure S1. Scatter plots of the first two principal components (PC1, PC2) of genetic ancestry for 1000 Genomes Project subjects and study participants. The 1000 Genomes Project subjects (*n* = 2,504) were labeled within five super-populations: African, European, East Asian, South Asian, and American. The study participants included in the genetic ancestry analysis (*n* = 141) were labeled with their genetic ancestry: African (*n* = 63), European (*n* = 51), South Asian (*n* = 13), East Asian (*n* = 7), or American (*n* = 7). Each data point represents one panelist.

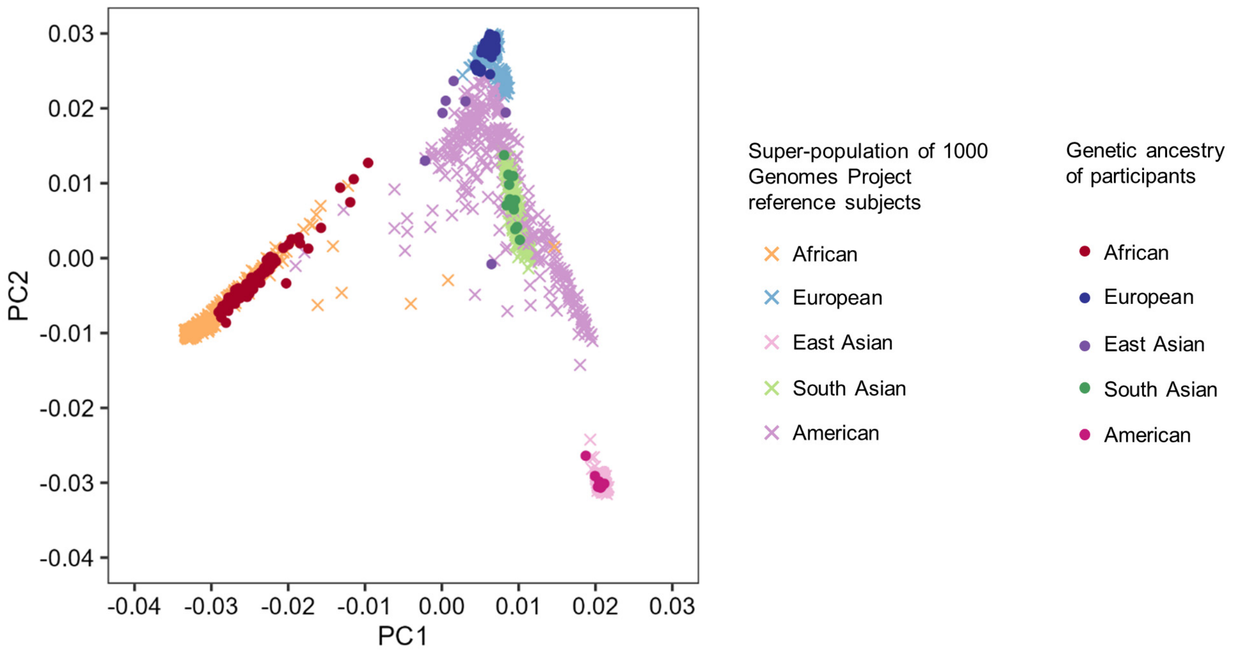

Figure S2. Relationship between panelists' first two principal components of genetic ancestry and their irritation, sweetness and hedonic ratings of Motrin™. Scatter plots of the first two principal components (PC1, PC2) and irritation (A-B), sweetness (C-D), and hedonic (E-F) gLMS ratings after swallowing Motrin™. Each data point represents one panelist (*n* = 141) labeled by their genetic ancestry: African (*n* = 63), European (*n* = 51), South Asian (*n* = 13), East Asian (*n* = 7), or American (*n* = 7). Because irritation ratings were not normally distributed, square root transformation was applied prior to analysis; however, original gLMS data are plotted to preserve the integrity of the meaning of the scale. *P* values were computed using linear regression between PC1 or PC2 and specified gLMS rating. Predicted gLMS ratings from linear regressions are indicated with a solid black line and the 95% confidence interval is indicated by the grey area.

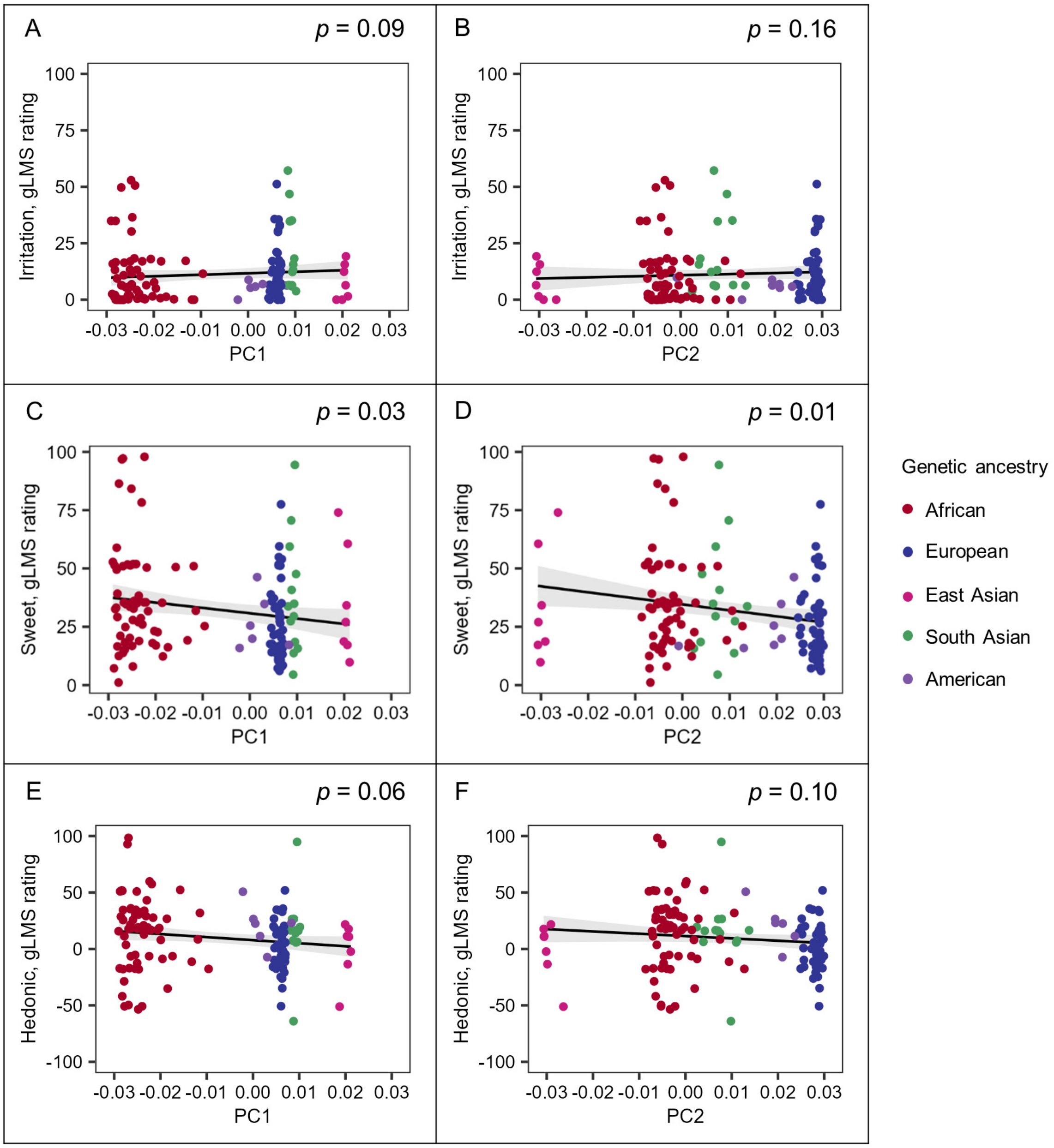

Figure S3. Relationship between the panelists' first two principal components of genetic ancestry and whether they experienced specific chemesthetic sensations. Boxplots of the first two principal components (PC1, PC2) of genetic ancestry of study participants and whether they experienced the urge cough or scratch (*n* = 63) or not (n = 78) (A-B) or whether they experienced tingling in throat (*n* = 49) or not (*n* = 92) (C-D) after swallowing Motrin™. Boxplots show the median value at the center line, the box spans the inter-quartile range, and the whiskers span the minimum and maximum (without outliers). Each data point represents one panelist (*n* = 141) labeled by their genetic ancestry: African (*n* = 63), European (*n* = 51), South Asian (*n* = 13), East Asian (*n* = 7), or American (*n* = 7). *P* values were computed using logistic regression between PC1 or PC2 and each chemosensory phenotype.

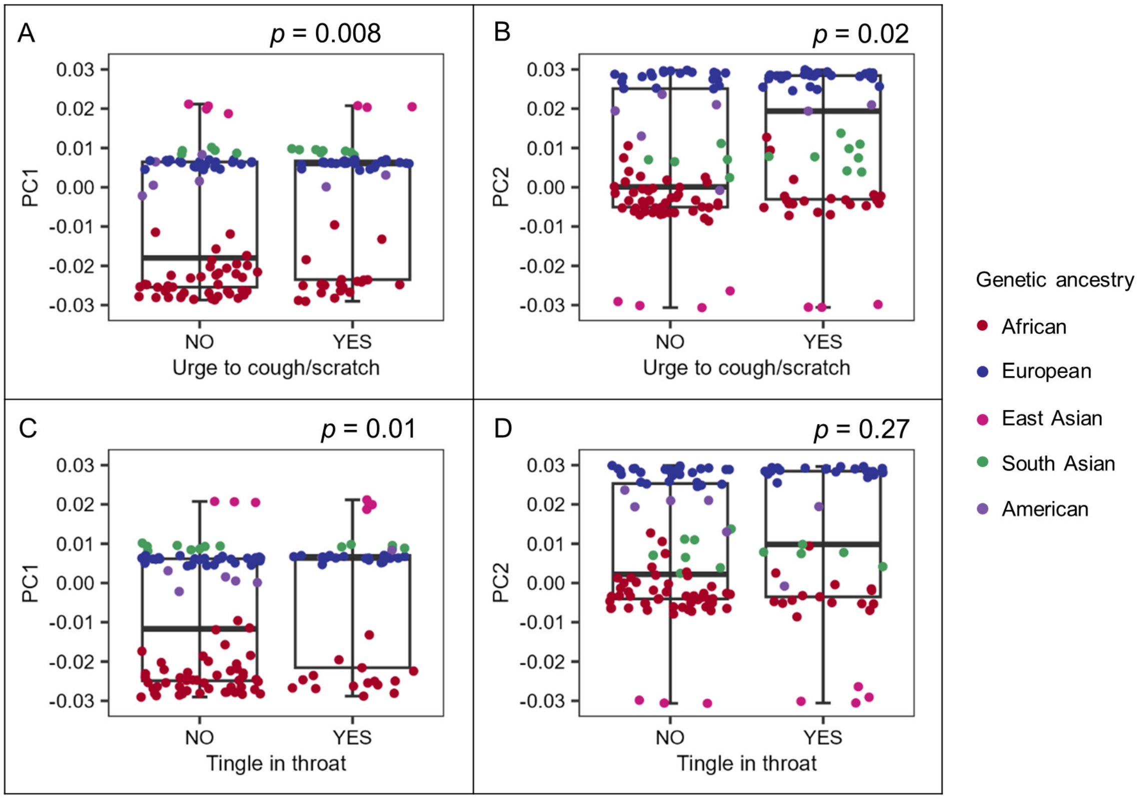

Supplement: Supplementary file 1 [file ijms-24-13050-s001.zip › ijms-2537088-supplementary.pdf]
